# Acquisition of Knowledge and Clinical Skills in Diagnosing Acute Appendicitis Among Medical Students: Contributing Factors and Overall Assessment

**DOI:** 10.7759/cureus.103051

**Published:** 2026-02-05

**Authors:** Mohamed E Salih, Abdullah M Alessa, Mohammed A Alqarni, Abdulkhaliq S Alqarni, Mohammed A Alrizqi, Abdulrahman M Alnashri

**Affiliations:** 1 Surgery, Al-Qunfudhah Medical College, Umm Al-Qura University, Al‑Qunfudhah, SAU; 2 Medicine, Al-Qunfudhah Medical College, Umm Al-Qura University, Al‑Qunfudhah, SAU

**Keywords:** appendicitis, diagnosis, emergency, knowledge, skill

## Abstract

Background and aim

Acute appendicitis (AA) is one of the most common surgical emergencies and serves as a key indicator of diagnostic proficiency among medical trainees. Variability in clinical presentations, the occurrence of atypical cases, and the ongoing evolution of diagnostic and management strategies can pose significant challenges for undergraduate medical students, particularly those with limited clinical exposure. This study aimed to assess the knowledge and applied clinical reasoning of fifth- and sixth-year medical students regarding the diagnosis and management of AA. The primary objective was to evaluate students’ understanding of classical and atypical clinical presentations, diagnostic approaches, complications, and management strategies. The secondary objective was to identify educational and exposure-related factors associated with variations in knowledge levels.

Methods

A cross-sectional study was conducted from April to August 2025, targeting fifth- and sixth-year medical students at Al-Qunfudhah Medical College. Using universal sampling, a semistructured questionnaire collected sociodemographic data and included 28 questions assessing knowledge and clinical skills related to appendicitis. Data were analyzed using IBM SPSS Statistics for Windows, Version 28.0 (Released 2021; IBM Corp., Armonk, NY, USA).

Results

A total of 100 medical students from Al-Qunfudhah Medical College participated; most were under 24 years of age (55%) and male (63%). Fifth-year students comprised 60% of the sample. Nearly half of the participants (48%) had encountered 10-20 appendicitis cases during clinical rotations. Participants demonstrated strong recognition of key clinical features, including continuous sharp pain (81%), periumbilical pain migration (77%), and right lower quadrant localization (74%). Knowledge gaps were identified in recognizing atypical presentations (56%) and pregnancy-related pain variations (56%). Awareness of diagnostic tools was highest for CT scans (86%) and inflammatory markers (69%), while knowledge of ultrasound (62%) and MRI (57%) was moderate. Students demonstrated good understanding of complications such as bleeding (91%) and abscess formation (83%) but showed less awareness of peritonitis (58%). While 76% preferred surgical management, only 55% supported medical management options. Overall, 49% demonstrated moderate knowledge, 24% high knowledge, and 27% low knowledge. Clinical exposure correlated significantly with higher knowledge levels (p = 0.049), whereas age, sex, and year of study showed no significant associations.

Conclusions

This study demonstrates that medical students at Al-Qunfudhah Medical College have satisfactory knowledge of the classical features and surgical treatment of appendicitis. However, gaps remain in their understanding of atypical presentations, diagnostic tools, and preoperative complications. While students were able to identify common symptoms and postoperative risks, their knowledge was less consistent when applied to varied clinical scenarios encountered in real-world settings.

## Introduction

Acute appendicitis (AA) remains one of the most prevalent surgical emergencies worldwide, affecting approximately 7-9% of the population, with a lifetime risk of approximately one in 11 individuals developing the condition [1]. Despite being a common and well-recognized disease, its diagnosis continues to present a clinical challenge owing to variable presentations, overlap with other abdominal conditions, and dependence on the clinician’s experience. Inaccurate or delayed diagnosis may lead to serious complications such as perforation, peritonitis, or sepsis, whereas overdiagnosis results in unnecessary surgical interventions and increased negative appendectomy rates [2].

The European Association for Endoscopic Surgery consensus emphasizes that accurate diagnosis of appendicitis requires a standardized approach combining clinical assessment, laboratory investigations, and imaging modalities [3]. However, evidence indicates that reliance on clinical evaluation alone can miss up to 24% of true appendicitis cases [1]. Studies have also identified cognitive biases, limited clinical experience, and overreliance on laboratory or imaging tests as major contributors to diagnostic errors in emergency settings [2]. These errors not only increase morbidity but also represent a significant medicolegal concern, with appendicitis ranking among the leading causes of diagnostic-related malpractice claims [2].

Appendicitis serves as a critical benchmark of diagnostic competence for medical students and junior trainees. Recognizing typical and atypical presentations, interpreting supportive laboratory findings such as leukocytosis and elevated CRP levels, and selecting appropriate imaging modalities (ultrasound, CT, or MRI) are essential clinical skills [1,3]. However, limited exposure to real-world cases and insufficient hands-on training may impair the ability to effectively apply theoretical knowledge in acute care settings.

Recent studies have explored this issue in medical education. Jaber (2024) assessed final-year medical students in Iraq and reported that, although theoretical knowledge of appendicitis was high, with 86.6% correctly identifying the disease, only 41.2% had encountered real cases during their training [4]. Furthermore, students demonstrated variable understanding of risk factors and complications, with notable gaps in identifying atypical or subclinical presentations [4]. These findings reflect global trends, showing that while students are generally competent in recognizing classical symptoms, such as pain migration to the right lower quadrant, many struggle to differentiate uncomplicated from complicated cases and to integrate imaging and laboratory data into diagnostic reasoning [1,3].

Diagnostic inaccuracy in appendicitis has important implications for patient safety and healthcare systems. Misdiagnosis occurs in approximately 6-7% of emergency presentations and is associated with preventable complications and prolonged hospital stays [2]. Therefore, strengthening undergraduate education in this area is critical for improving early recognition, reducing diagnostic delays, and minimizing negative appendectomy rates.

Accordingly, this study aimed to assess the acquisition of knowledge and clinical skills in diagnosing AA among medical students and to identify contributing factors affecting diagnostic performance. By evaluating both theoretical understanding and applied clinical reasoning, the findings of this study may help inform future curriculum development and enhance diagnostic training in surgical and emergency medicine.

## Materials and methods

Study design and setting

A cross-sectional study was conducted in Saudi Arabia from April 2025 to August 2025, targeting fifth- and sixth-year medical students at Al-Qunfudhah Medical College. A universal sampling approach was used.

Sample size

The sample size was calculated to determine the minimum number of responses required for the sample to be representative of the target population. The Raosoft sample size calculator (Raosoft Inc., Seattle, WA, USA) was used, assuming a response distribution of 50%, a margin of error of 5%, and a confidence level of 95%. The required minimum sample size was calculated using the following formula:

$n = \frac{Z_{\alpha}^2 \times P \times (1 - P)}{d^2},$

where n represents the required sample size, Zα represents the Z-value corresponding to the desired confidence level (1.96 for a 95% confidence level), P represents the estimated proportion of the attribute present in the population (0.50), and d represents the margin of error (0.05). Based on this calculation, the minimum required sample size was 100 participants.

Inclusion and exclusion criteria

Participants were eligible if they were actively enrolled in the fifth or sixth year of the medical program and had completed the general surgery rotation. Students who had not yet completed the relevant clinical rotations were excluded.

Data collection

The study utilized a semistructured questionnaire adapted from a relevant study. The questionnaire consisted of five sections. The first section collected sociodemographic characteristics. The second section assessed participants’ knowledge of symptoms and diagnosis of appendicitis. The third section focused on investigation methods used to diagnose appendicitis. The fourth section evaluated participants’ knowledge of appendicitis-related complications. The fifth section assessed knowledge of appendicitis management. The final version of the questionnaire comprised 33 questions across five sections.

Scoring system

A total of 33 statements were used to assess participants’ knowledge and clinical skills related to appendicitis: five items for demographic information, 10 items assessing knowledge of symptoms and clinical features, and 18 items evaluating knowledge of diagnosis and management. One point was awarded for each correct answer, while zero points were assigned for incorrect responses or selections of “I don’t know.”

Likert scales (three-point and five-point) were used for scoring. Participants were categorized into three groups based on their total scores, which ranged from 0 to 28 points. Scores of 12 or below were classified as a low level of knowledge, scores between 13 and 17 as a moderate level of knowledge, and scores of 18 or above as a high level of knowledge. In this study, the term “clinical skills” refers to applied clinical reasoning and decision-making abilities as assessed through scenario-based and knowledge-application questionnaire items. These items evaluated students’ ability to interpret clinical features, select appropriate diagnostic modalities, recognize complications, and choose management strategies, rather than direct observation of procedural or bedside skills.

Statistical analysis

All data were analyzed using IBM SPSS Statistics for Windows, Version 28.0 (Released 2021; IBM Corp., Armonk, NY, USA). Descriptive statistics were used to summarize categorical variables, including frequencies and percentages. For inferential analysis, the chi-square test (Pearson’s χ²) was applied to examine associations between students’ knowledge levels and demographic or academic characteristics. Exact probability tests were used when small cell counts were present. A p-value of less than 0.05 was considered statistically significant. Data cleaning and coding were performed prior to analysis to ensure completeness and consistency.

## Results

Table 1 presents the sociodemographic and academic characteristics of the 100 medical students who participated in the study at Al-Qunfudhah Medical College. The age distribution showed that 55 students (55.0%) were younger than 24 years, while 45 students (45.0%) were 24 years or older. Regarding sex, male students predominated, with 63 participants (63.0%) compared with 37 female students (37.0%). Most participants were in their fifth year of medical school (60.0%), while the remaining 40 students (40.0%) were in their sixth year. With respect to clinical exposure to appendicitis cases during rotations, 35 students (35.0%) reported encountering fewer than 10 cases, 48 students (48.0%) reported exposure to 10-20 cases, and 17 students (17.0%) reported exposure to more than 20 cases.

**Table 1:** Sociodemographic and academic characteristics of the medical students at Al-Qunfudhah Medical College (N = 100)

Demographics	N	%
Age (years)		
<24 years	55	55.0%
>24 years	45	45.0%
Gender		
Male	63	63.0%
Female	37	37.0%
Year of medical study		
Fifth year	60	60.0%
Sixth year	40	40.0%
Number of appendicitis cases encountered during rotations		
<10 cases	35	35.0%
10-20 cases	48	48.0%
>20 cases	17	17.0%

Table 2 shows that a large majority of students correctly identified key clinical features of AA. Eighty-one students (81.0%) recognized that appendicitis pain is often continuous and sharp, and 77 students (77.0%) correctly identified that initial pain typically begins in the periumbilical region. In addition, 74 students (74.0%) were aware that the pain later shifts to the right lower quadrant. However, knowledge was less consistent in certain areas. Only 56 students (56.0%) correctly reported that appendicitis may present without clear clinical symptoms, and the same proportion (56.0%) recognized that pain location may differ during pregnancy due to anatomical changes. While 80 students (80.0%) correctly associated loss of appetite with appendicitis, fewer recognized the relevance of digestive symptoms (65.0%) or the visceral nature of early pain (70.0%).

**Table 2:** General knowledge of medical students regarding the clinical presentation and recognition of AA (N = 100) AA, acute appendicitis

General knowledge	Yes	No	I don’t know
Does AA often present without clear clinical symptoms?	56 (56.0%)	42 (42.0%)	2 (2.0%)
Is the pain of appendicitis often described as continuous, sharp pain?	81 (81.0%)	18 (18.0%)	1 (1.0%)
Initially, is the pain of appendicitis localized to the periumbilical region?	77 (77.0%)	21 (21.0%)	2 (2.0%)
Does the pain eventually shift to the lower right side of the abdomen?	74 (74.0%)	18 (18.0%)	8 (8.0%)
Does the pain radiate internally or follow a visceral pattern?	70 (70.0%)	15 (15.0%)	15 (15.0%)
In pregnant individuals, does the location of the pain differ due to anatomical changes?	56 (56.0%)	24 (24.0%)	20 (20.0%)
Is abdominal pain commonly described as a key symptom by patients with appendicitis?	73 (73.0%)	17 (17.0%)	10 (10.0%)
Are digestive issues such as nausea, vomiting, indigestion, or abdominal discomfort typical symptoms of appendicitis?	65 (65.0%)	26 (26.0%)	9 (9.0%)
Is the diagnosis of appendicitis primarily clinical rather than investigation-based?	72 (72.0%)	19 (19.0%)	9 (9.0%)
Is loss of appetite frequently associated with appendicitis?	80 (80.0%)	16 (16.0%)	4 (4.0%)

Table 3 summarizes students’ knowledge and perceptions of diagnostic methods for appendicitis. Most students agreed or strongly agreed that blood tests evaluating WBC counts (64.0%) and neutrophil levels (70.0%) are important diagnostic tools. Similarly, 69.0% agreed or strongly agreed that inflammatory markers, such as CRP and erythrocyte sedimentation rate, are useful. Regarding imaging modalities, CT scans received the highest level of support, with 86 students (86.0%) agreeing or strongly agreeing with their diagnostic value. Ultrasound was also positively recognized by 62 students (62.0%). In contrast, abdominal X-rays were less favored, with only 31 students (31.0%) agreeing or strongly agreeing with their usefulness and 50 students (50.0%) expressing disagreement. Opinions regarding MRI were variable, with 57 students (57.0%) agreeing or strongly agreeing with its utility, while 36 students (36.0%) disagreed or strongly disagreed.

**Table 3:** Medical students’ knowledge and perceptions of diagnostic methods for appendicitis (N = 100)

Diagnostic method	Strongly disagree	Disagree	Neither	Agree	Strongly agree
Blood test: evaluation of WBC levels	21 (21.0%)	13 (13.0%)	2 (2.0%)	31 (31.0%)	33 (33.0%)
Blood test: assessment of neutrophil levels	19 (19.0%)	8 (8.0%)	3 (3.0%)	44 (44.0%)	26 (26.0%)
Blood test: inflammatory markers (CRP and erythrocyte sedimentation rate)	24 (24.0%)	7 (7.0%)	0 (0.0%)	31 (31.0%)	38 (38.0%)
Imaging: abdominal X-ray	21 (21.0%)	29 (29.0%)	19 (19.0%)	12 (12.0%)	19 (19.0%)
Ultrasound examination	17 (17.0%)	14 (14.0%)	7 (7.0%)	31 (31.0%)	31 (31.0%)
CT scan	5 (5.0%)	7 (7.0%)	2 (2.0%)	27 (27.0%)	59 (59.0%)
MRI	29 (29.0%)	7 (7.0%)	7 (7.0%)	34 (34.0%)	23 (23.0%)

Table 4 presents students’ knowledge regarding common complications of appendicitis before and after surgery. Most students correctly identified major preoperative complications, including appendicular abscess (83.0%) and visceral perforation (75.0%), while awareness of peritonitis was comparatively lower (58.0%). Postoperative complications were more widely recognized, with high proportions identifying bleeding (91.0%) and surgical wound infection (87.0%) as potential outcomes. Knowledge of postoperative adhesions was also relatively strong (76.0%); however, only 61.0% correctly identified small bowel obstruction as a possible postoperative complication.

**Table 4:** Medical students’ knowledge of appendicitis complications before and after surgery (N = 100)

Complication	Yes	No	I don’t know
Before surgery			
Visceral perforation	75 (75.0%)	21 (21.0%)	4 (4.0%)
Peritonitis	58 (58.0%)	32 (32.0%)	10 (10.0%)
Appendicular abscess	83 (83.0%)	9 (9.0%)	8 (8.0%)
After surgery			
Surgical wound infection	87 (87.0%)	8 (8.0%)	5 (5.0%)
Bleeding	91 (91.0%)	3 (3.0%)	6 (6.0%)
Adhesions	76 (76.0%)	14 (14.0%)	10 (10.0%)
Small bowel obstruction	61 (61.0%)	27 (27.0%)	12 (12.0%)

Table 5 outlines medical students’ knowledge and perceptions of different treatment approaches for appendicitis. A strong majority of students (79.0%) agreed or strongly agreed that all patients should be started on antibiotics. However, views on medical management as the preferred approach were more divided, with 55.0% agreeing or strongly agreeing and 40.0% disagreeing or strongly disagreeing. In contrast, most students (76.0%) supported the view that appendicitis is best managed surgically, reflecting confidence in the standard surgical approach. Additionally, 69.0% of students agreed or strongly agreed that laparoscopic appendectomy is associated with a shorter hospital stay and faster recovery.

**Table 5:** Medical students’ knowledge and perceptions of treatment approaches for appendicitis (N = 100)

Statement	Strongly disagree	Disagree	Neither	Agree	Strongly agree
Starting all patients on antibiotics	13 (13.0%)	6 (6.0%)	2 (2.0%)	36 (36.0%)	43 (43.0%)
Medical management is the best approach for managing appendicitis	27 (27.0%)	13 (13.0%)	5 (5.0%)	37 (37.0%)	18 (18.0%)
Appendicitis is best managed surgically	17 (17.0%)	7 (7.0%)	0 (0.0%)	20 (20.0%)	56 (56.0%)
Laparoscopic appendectomy offers a shorter hospital stay and faster recovery	18 (18.0%)	9 (9.0%)	4 (4.0%)	24 (24.0%)	45 (45.0%)

**Figure 1:**
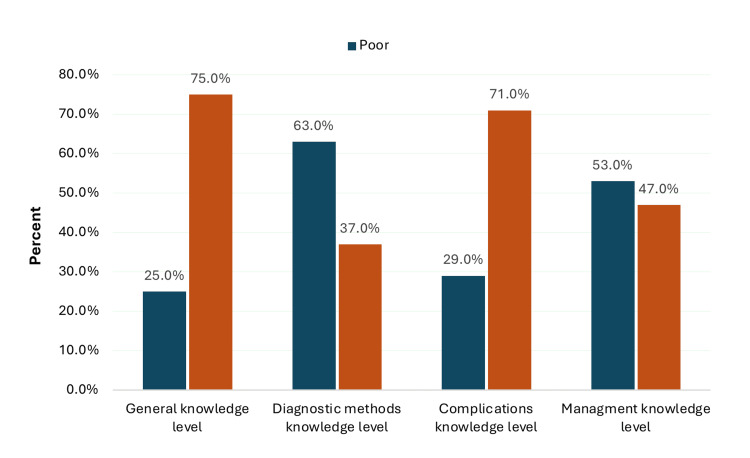
Overall levels of knowledge among medical students regarding appendicitis by domain (N = 100)

Figure 1 illustrates the distribution of medical students’ knowledge levels regarding appendicitis across four key domains: general knowledge, diagnostic methods, complications, and management. The highest proportion of students demonstrated good knowledge in the general knowledge domain (75.0%), followed by the complications domain (71.0%). In contrast, knowledge levels were lower in the diagnostic methods and management domains, with only 37.0% and 47.0% of students, respectively, achieving a good knowledge level. Notably, 63.0% of students demonstrated poor knowledge of diagnostic methods, and 53.0% had poor knowledge related to management strategies.

**Figure 2:**
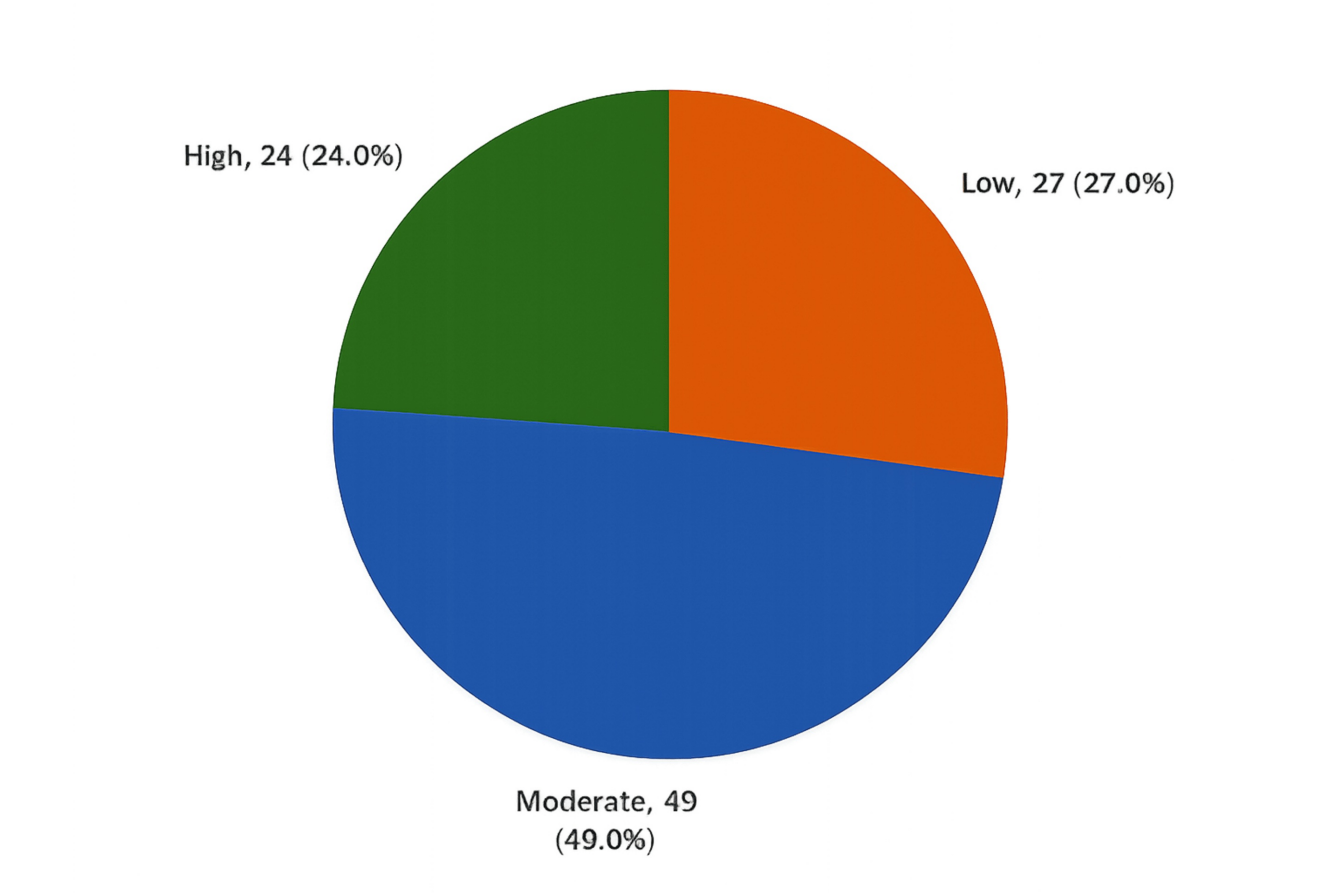
Overall knowledge level of medical students regarding appendicitis at Al-Qunfudhah Medical College (N = 100)

Figure 2 presents the overall knowledge level of medical students regarding appendicitis at Al-Qunfudhah Medical College. The majority of students demonstrated a moderate level of knowledge (49.0%). High knowledge levels were observed in 24.0% of students, while 27.0% had a low level of knowledge.

Table 6 examines the relationship between demographic and academic factors and the overall knowledge level of medical students regarding appendicitis. Among all variables analyzed, only clinical exposure to appendicitis cases during rotations demonstrated a statistically significant association with knowledge level (p = 0.049). Students who had encountered 10-20 cases showed the highest proportion of high knowledge levels (33.3%), whereas those with fewer than 10 cases were more likely to have low knowledge levels (40.0%). Students who had encountered more than 20 cases predominantly fell within the moderate knowledge category (70.6%), with a smaller proportion demonstrating high knowledge (11.8%). In contrast, age (p = 0.434), sex (p = 0.372), and year of study (p = 0.330) were not significantly associated with knowledge levels. Although a higher proportion of female students (35.1%) than male students (22.2%) fell into the low knowledge category, and sixth-year students (35.0%) demonstrated a higher proportion of low knowledge compared with fifth-year students (21.7%), these differences were not statistically significant.

**Table 6:** Factors associated with medical students’ knowledge of appendicitis and preparedness

Factors	Overall knowledge level						p-Value
	Low		Moderate		High		
	N	%	N	%	N	%	
Age (years)							0.434
<24 years	12	21.80%	29	52.70%	14	25.50%	
>24 years	15	33.30%	20	44.40%	10	22.20%	
Gender							0.372
Male	14	22.20%	33	52.40%	16	25.40%	
Female	13	35.10%	16	43.20%	8	21.60%	
Year of medical study							0.330
Fifth year	13	21.70%	32	53.30%	15	25.0%	
Sixth year	14	35.0%	17	42.50%	9	22.50%	
Number of appendicitis cases encountered during rotations							0.049
<10 cases	14	40.0%	15	42.90%	6	17.10%	
10-20 cases	10	20.80%	22	45.80%	16	33.30%	
>20 cases	3	17.60%	12	70.60%	2	11.80%	

## Discussion

In this study, most participating medical students were under the age of 24 and predominantly male. The majority were in their fifth year of medical school, indicating that they were still in the earlier stages of their clinical training. In terms of practical exposure, students varied in their experience with appendicitis cases during clinical rotations. A considerable number reported only limited exposure to actual cases, while fewer students reported having encountered a higher number of cases.

Regarding students’ knowledge and preparedness, the findings indicate that most students have a good understanding of the typical clinical features of AA. This reflects the effective delivery of surgical education at the undergraduate level and suggests that students are well prepared to recognize standard presentations of the condition. However, the study also highlights notable deficiencies in clinical readiness. Many students demonstrated limited awareness of atypical or subclinical presentations, which is concerning. These less common presentations are known to contribute to diagnostic delays and poorer outcomes, particularly in high-risk groups in whom classical signs may be absent [5,6]. Additionally, knowledge regarding variations in the presentation of appendicitis in specific physiological states, such as pregnancy, was inconsistent among participants. This is clinically significant because anatomical and physiological changes during pregnancy can substantially alter symptom patterns, and appendicitis remains one of the most common nonobstetric surgical emergencies in pregnant patients [7,8]. Overall, while students appear confident in identifying typical cases, their preparedness for complex and variable clinical scenarios remains limited.

With respect to diagnostic modalities, the findings show that medical students generally have a good understanding of diagnostic tools for appendicitis, consistent with current clinical guidelines. Most students correctly recognized the role of laboratory investigations, including WBC count, neutrophil percentage, and CRP, as supportive tools for diagnosis and assessment of inflammatory activity [9,10]. There was also strong awareness of the value of CT, which is considered the gold standard for diagnosing appendicitis in nonpregnant adults because of its high diagnostic accuracy [11].

Students also demonstrated practical awareness of ultrasound, which is particularly relevant as a first-line imaging modality in children and pregnant women, where radiation exposure should be avoided [12]. However, knowledge regarding MRI was less consistent. This may reflect its limited availability and use in emergency settings, despite its proven diagnostic accuracy, particularly during pregnancy [13]. Importantly, many students disagreed with the diagnostic value of abdominal X-rays, which is appropriate given their low sensitivity and limited role in the diagnosis of appendicitis in current practice [14]. Nevertheless, a proportion of students still perceived X-rays as useful, possibly reflecting outdated teaching or limited familiarity with contemporary diagnostic guidelines.

In terms of knowledge of complications, the study demonstrated strong awareness of common postoperative risks but comparatively weaker recognition of serious preoperative complications associated with delayed diagnosis. Most students correctly identified postoperative complications such as bleeding and surgical site infection, which are routinely emphasized in surgical education and postoperative care [15]. Knowledge of adhesions and small bowel obstruction also suggests an adequate understanding of long-term outcomes following abdominal surgery [16]. However, insufficient knowledge was observed regarding preoperative complications, particularly peritonitis, which was less frequently recognized compared with complications such as appendicular abscess and visceral perforation. This is concerning, as peritonitis is a serious and potentially life-threatening consequence of untreated or delayed appendicitis [17]. Lower recognition of peritonitis may indicate limited understanding of the pathophysiological progression from localized inflammation to perforation and generalized infection. The finding that more students identified abscess formation as a localized complication rather than generalized peritonitis supports this interpretation [18].

Findings related to students’ understanding of treatment options for appendicitis reflect current clinical practices and ongoing debates in the field. Most students supported the initiation of antibiotic therapy as part of management, which aligns with current guidelines emphasizing early antibiotic administration to reduce septic complications, regardless of whether surgical intervention is ultimately performed [19]. This suggests a solid understanding of essential perioperative care principles. Variation in opinions regarding nonsurgical (medical) management mirrors real-world uncertainty and evolving clinical practice. While some students agreed with medical management as a first-line option, others disagreed, reflecting the ongoing debate regarding antibiotic-only treatment for uncomplicated appendicitis [20]. This variability suggests exposure to contemporary clinical evidence rather than reliance solely on traditional approaches. It also highlights the complexity of balancing nonoperative management against concerns related to recurrence and long-term outcomes [21]. Despite this, a strong majority of students continued to favor surgical management, confirming that appendectomy remains widely accepted as the standard of care. Furthermore, high awareness of the benefits of laparoscopic appendectomy, including faster recovery and shorter hospital stays, indicates that students are well informed about modern minimally invasive surgical techniques that are now standard in many healthcare settings [22].

Overall, the knowledge level of medical students regarding appendicitis indicates that while many demonstrate a moderate level of understanding, a substantial proportion continue to exhibit limited knowledge. This suggests that although foundational concepts are being taught, some students may lack the depth of knowledge required for confident and effective clinical decision-making, particularly in urgent situations such as the evaluation of acute abdominal pain. When compared with previous research, similarities and differences can be observed [4]. Both studies reported a solid foundational understanding of appendicitis; however, our findings reveal gaps in recognizing atypical presentations, preoperative complications, and symptom variations, including those occurring during pregnancy. Clinical exposure was limited in both studies. Awareness of imaging modalities, particularly CT and ultrasound, was high in both cohorts, whereas MRI remained less well understood. Surgical management was widely accepted, with laparoscopic appendectomy slightly preferred. Notably, our study demonstrated broader awareness of nonoperative management strategies, suggesting increasing exposure to evolving treatment guidelines. Additionally, the satisfactory awareness of appendicitis (86.6%) and perforated appendicitis (78.2%) aligns with studies emphasizing the importance of theoretical teaching in medical curricula [23]. Similarly, Abourashid et al. [24] reported an acceptable level of awareness among medical students at both Syrian Private University and Damascus University. There remains a relative scarcity of published literature assessing awareness of this surgical condition among medical students. In a Saudi Arabian study evaluating awareness of acute abdominal pain among medical students, the reported awareness level was 23.4, representing an average degree of knowledge [25].

Strengths and limitations

This study has several strengths. It addresses a clinically relevant and high-risk surgical condition with important implications for patient safety and undergraduate medical education. The inclusion of multiple knowledge domains, such as clinical presentation, diagnostic modalities, complications, and management strategies, allowed for a comprehensive assessment of students’ preparedness. In addition, analysis of exposure-related factors, particularly clinical case volume during rotations, provided valuable insight into the role of experiential learning in knowledge acquisition.

However, several limitations should be acknowledged. First, the study was conducted at a single medical college, which may limit the generalizability of the findings to other institutions or educational settings. Second, the use of a self-reported questionnaire introduces the possibility of response and recall bias. Third, although applied clinical reasoning was assessed through scenario-based and knowledge-application questions, the study did not include objective or performance-based evaluations of clinical or procedural skills, such as objective structured clinical examinations or simulation-based assessments. Finally, the cross-sectional design limits the ability to infer causal relationships between educational exposure and knowledge outcomes. These limitations underscore the need for future multicenter studies incorporating objective assessment tools and longitudinal designs.

## Conclusions

This study demonstrates that medical students at Al-Qunfudhah Medical College have a satisfactory level of knowledge regarding the classical features and surgical management of appendicitis. However, notable gaps remain in their understanding of atypical presentations, the full range of diagnostic modalities, and preoperative complications. While most students were able to identify common symptoms and postoperative risks, their knowledge was less consistent when addressing the varied clinical scenarios encountered in real-world practice. Knowledge levels were strongest in the domains of general symptoms and complications but weaker in diagnostic approaches and management strategies.

Furthermore, increased clinical exposure to appendicitis cases was the only factor found to be statistically significantly associated with higher knowledge levels. Based on these findings, greater emphasis on case-based learning within the undergraduate curriculum is recommended, particularly focusing on nonclassical presentations and diagnostic reasoning. In addition, expanding opportunities for clinical exposure through simulation-based training, extended clinical rotations, or virtual case discussions may further enhance students’ preparedness for managing AA.
